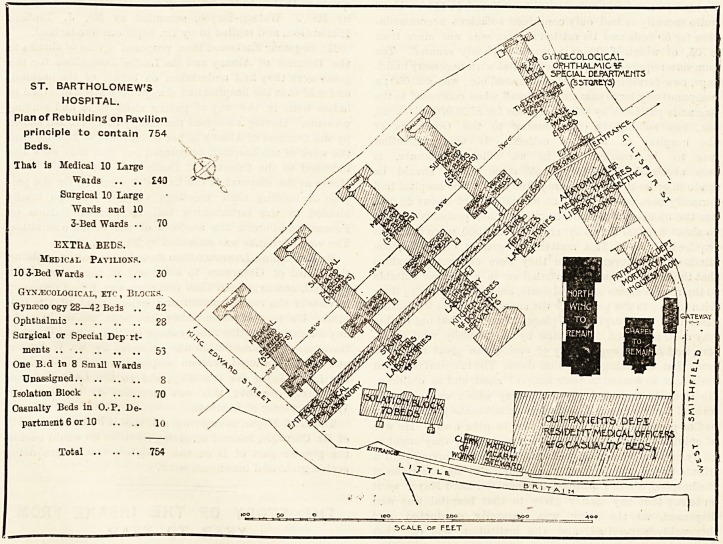# St. Bartholomew's Hospital

**Published:** 1904-01-23

**Authors:** 


### HOSPITAL ADMINISTRATION.

### CONSTRUCTION AND ECONOMICS.

### ST. BARTHOLOMEW'S HOSPITAL.

#### THE PRESENT POSITION.

A PLEA FOR UNANIMITY.

WE publish elsewhere a letter issued to the Press by Sir Trevor Lawrence, the Treasurer of Bart's. These letters have caused much discussion, seeing that Sir Trevor admits that the authorities had realised for a long time that the original scheme for reconstructing the hospital could be improved upon. Yet Sir Trevor is content to go to the public with an appeal for funds without a completed and perfected plan for rebuilding the hospital, and without the names of the Governors and members of the Medical Staff, who will form the special building committee who are to be charged with the responsibility of producing a perfected plan for, a modern hospital. Again, Sir Trevor insists, that if the Bart's. appeal were again deferred, it would involve the waste of a second year's interest on the purchase money of the new land. Every business man knows, however, that any scheme for the purchase of land in the City, nowadays, necessitates a provision for adding, to prime cost, say, five years' interest on the purchase money, to cover the period required for the development of the land and the erection of buildings upon it. In the case of Bart's. Hospital, the surest way of expediting the erection of an entirely new hospital is, to base the appeal for money upon a perfected and inclusive plan, which will guarantee the public, that the total sum asked for will be adequate and final so far as St. Bartholomew's is concerned. To issue an appeal until these guarantees are provided will only tend, to weaken its effect; to necessitate continuous efforts to raise money for the new buildings during a number of years; and so to reduce St. Bartholomew's Hospital to the unenviable position of joining the ranks of the chronic beggars, although the total value of its properties amounts in round numbers to £4,000,000 sterling. Stated thus the position reveals a want of apprehension of the requirements precedent to the issue of an appeal, which on its merits might otherwise win the support of all who are able to contribute to it. It is not a question of delaying the appeal, for no really successful appeal can be launched until someone has organised efficiently the vast army of influential people whom Bart's. should be able to enlist as workers in the present emergency.

If matters are allowed to rest as they stand to day there is good reason to fear that the meeting at the Mansion House will reveal diversities of opinion which cannot fail to injure the chances of the proposed appeal. We have been working continuously since the last meeting of Governors in the hope of uniting all the forces which gather round St. Bartholomew's Hospital. In furtherance of this object the following basis of agreement may, we hope, be adopted by the Treasurer and the acting Medical Staff, when it would be accepted by everybody.

Our basis has the further advantage that the wise words of the President, H.R.H. the Prince of Wales, would so receive the respectful treatment their wisdom merits. So far the Bart's. authorities appear to have ignored the Prince's warning.

A BASIS OF AGREEMENT.

We have shown that there is no perfected plan, and the treasurer makes it clear that there is not time to prepare such a plan before the meeting on Tuesday next, the 26th inst. By that date it would certainly be impossible to prepare a document embodying the guarantees and full descriptions we have referred to, with plans. Yet without them any appeal must fail to commend itself to the fullest sympathy of the public. How can these two essential conditions of success be provided for by the authorities so as to satisfy all parties at Tuesday's meeting? Let the Treasurer in his opening statement announce the names of the special building committee of Governors and members of the Medical Staff who will be charged with the responsibility of producing a perfected plan. Let him also name at least half-a-dozen members of the Appeal Committee with power to add to their number, and let these six gentlemen include some at least who are known to possess experience in the art of raising large sums of money for public purposes. Let him point out that the organisation of the necessary machinery and the preparation of the plans will take probably two months at least, and in consequence it is desirable to leave the date of issue and the actual wording of the appeal to be settled by the Appeal Committee in consultation with the Lord Mayor. These proposals are very simple, but they are practical and businesslike, and from an intimate knowledge of the inner workings and opinions of the various parties immediately concerned, we know, that if the treasurer adopts them, he may rely upon the united support of everybody who has the best interests of St. Bartholomew's Hospital at heart.

No doubt, as a writer in the *Times* insists, "the proper time for urging Bart's. claims is the time when they arise, the time when, in other words, if they are not satisfied, the work of the hospital will be less completely accomplished than it ought to be." This is true, no doubt, but the time cannot be said to have arrived for the issue of an appeal, until all the data for making that appeal effective are ascertained and known. We all can agree "that the position of St. Bartholomew's as the special hospital *par excellence* gives it a claim upon the City which cannot be gainsaid," and that "it would be little short of a disgrace to the wealthy men of that pre-eminently wealthy square mile, if their hospital were not maintained in a condition rivalling, if not excelling, that of any other in the world." But before those referred to can be made responsible for the failure of an appeal, it is essential that the scheme on which it is based should afford the business men referred to, full guarantees for excellence, completeness, and business management. In the absence of such evidence, no one of them would be justified in putting money into any scheme of business or charity. Hence we ask the treasurer to at once supply the condition precedent to success, *i.e.*, the guarantees which can be provided in the form we have suggested.

### HOW TO PROVIDE BART'S. WITH MODERN HOSPITAL BUILDINGS.

### A Question of Plans.

In The Hospital of October 31st, 1903, pages 87 to 90, we published an article containing a model plan on the pavilion principle, for the rebuilding of St. Bartholomew's Hospital on an 8-acre site at Smithfield. We hope those of our readers who are interested in the important question which underlies the difficult problem of how to best utilise the 6½ acres of land to which the Bart's. site is now restricted by the action of the Post Office, will compare the plan on page 88 of The Hospital of October 31st last with the further plans which we publish in this issue.

In order to understand the present position and to form a judgment upon its possibilities, it must be assumed that the Post Office will make a private road some 45 feet wide to the whole extent of the southern boundary of St. Bartholomew's hospital site extending from King Edward Street to Giltspur Street. It is further understood that the architects of the hospital and the Post Office will co-operate together, to provide for the freest access of light and air to the hospital buildings, so that the 6½ acres occupied by St. Bartholomew's Hospital will be, in fact, a self-contained site, surrounded by streets and the Post Office road, with the advantage of the open area of Smithfield. It has been determined to remove the Nurses' Home and Medical College to new sites adjacent to that on which the hospital at present stands, and here it may be well to mention that a grave misapprehension appears to prevail on the subject, for we have reason to believe that it will be possible to erect the Nurses' Home in such a position as to enable the nurses to pass freely from the hospital to the home without traversing the public streets, without exposure to the weather, and without any appreciable loss of time in the journey. As the writer to the *Times* properly insists, considerations of superficial and cubic space, though essential, are not the only considerations to be borne in mind when planning a hospital, for the methods of construction and the freedom and distribution of the air supply are at least equally important.

### The Plans as Amended to Altered Conditions.

The writer in the *Times* claims "that the plans for the new hospital intended to take the place of the old St Bartholomew's will be of such a kind as not only to fulfil every sanitary requirement, but also to furnish every facility that science can require for the treatment of the sick." With a view to enable our readers to form their own judgment as to the extent to which the claim here advanced has been fulfilled in the plans submitted to the acting medical staff, we think it desirable to reproduce the revised draft plans, now under consideration, which embody the architect's proposals for the present site, omitting the Nurses' Home and the Medical College buildings. Sir Trevor Lawrence states that these plans have been carefully revised, and that the block plans show how the hospital buildings in the future may be distributed in more ways than one.

We propose to reproduce two of these block plans, Nos. 7 and 9, both dated January 14th, 1904, and signed by the hospital architect, Mr. E. B. I'Anson, M.A., F.R.I.B.A. A third plan was submitted, No. 8, which retained the existing east, south, and west

### PLAN No. 7.

This plan shows the existing buildings, which are to remain, in black. The new buildings are shown shaded, and the existing east, west, and south wings, and the church which will be demolished are shown by dotted lines. It will be observed that the kitchen is placed in connection with the out-patients' block, that it is far removed from the buildings containing the patients, and that, in consequence, very few, if any hot dinners, could ever be served in practice. It will further be seen that blocks F and E are so planned that the best aspect is destroyed by the situation of the sanitary and other blocks connected with them; the same may be said of blocks C and D, so that out of six new pavilions containing wards four may be said to be unsatisfactory. We hold that to connect all the pavilions by subways is, to say the least, not an ideal wings which were cut down so as to have a single instead of back to back wards on each floor, reducing the number of beds they contain to 272, and adding three ward pavilions to contain 335 beds, in addition to an isolation pavilion with 70 beds and 10 casualty beds in connection with the surgery. There was also provided a pathological department, the necessary houses, and accommodation for residents; the total number of beds being 687, and the estimated cost about £400,000. This scheme, however, involved the making of subways connecting all the wings and pavilions, and the erection of five operation theatres on the top of the north wing. The out-patient department and kitchen were placed as shown in plans 7 and 9 which follow.

arrangement, especially as the isolation pavilion is connected with the subway system, so that on hygienic grounds, as experience teaches, there are grave objections to the scheme. The plan also embodies the extraordinary proposal to place five new operating theatres on the top of the north wing which would entail that every patient to be operated upon must first be taken in a lift from the ward in the pavilion where he may be, and then through a number of subways to block H, that he must there be put into another lift and carried to the top of that building. After the operation the process must be reversed in the case of every patient operated upon, with consequences which, in practice, could not be unattended by danger and risk, which ought to be avoided in a modern hospital. We have not space to enter into a detailed criticism, but we may add that the plan provides for 704 beds, and that the estimated cost in round numbers we understand is about £370,000.

### PLAN No. 9.

This plan provides for the rebuilding of the whole hospital with the exception of the medical school buildings and library, and the ancient gateway. The new buildings and existing wings are shown as in the previous plan. It will be noticed that the kitchen is again divorced from the hospital proper, which would in practice probably make the new Bart's. the most unpopular hospital in London with its patients. The whole of the buildings are again connected up with subways and we need not repeat the objections. The ward pavilions will occupy the east and west sides of what may be described as the main street, which would be about 100 feet wide. The best aspect for the wards in blocks D, E, F, and also in the isolation block, is destroyed by the position in which the sanitary, etc., blocks, are placed, apparently in order to make the main street straight. The isolation block appears to be placed so near King Edward Street as to constitute an infringement of the Acts affecting the building of hospitals for infectious diseases, and if these conditions were to be fulfilled by setting back this block, it seems that the whole plan would be destroyed, so far as the present arrangements of the ward pavilions are concerned. We do not therefore propose to criticise this plan in detail.

Looking at plans 7 and 9 from the administrator's point of view, and remembering that Bart's. has a large medical school, we think, both in regard to economy of site and economy of administration, that it is a mistake to scatter ward blocks about the site without having any simple means of intercommunication. In the absence of such means, proper control, the enforcement of discipline, and the reduction of the cost per bed to a reasonable sum, may prove impossible. It is perfectly certain that a hospital erected on this "dog kennel" principle does not make the best of a town site, does not facilitate the work of the staff, neither does it promote the efficiency and the easy working of a great clinical hospital.

### A New St. Bartholomew's Upon the Pavilion Principle.

We will now give a plan which we have prepared, which fulfils all the requirements of the acting Medical Staff, and would provide a hospital on the pavilion principle to contain 754 beds. It will be instructive and helpful if the reader will compare this plan with the model plan published on page 88 of The Hospital of October 31st last; such a comparison will show at once how difficult it is to make the best of a restricted site, especially if it have a diameter which tends to decrease in length as in the case of Bart's. site. In the model 8-acre plan the diameter was wide enough to enable the pavilion to be readily swung in crescentic fashion. The 6½-acre site renders this method rather difficult of execution as the plan we publish to-day shows.

The pavilion principle has this great advantage over the dog-kennel principle adopted in plan No. 9, page 301, that it provides that :—

1. The wards shall occupy the best aspect, that they shall be in a self-contained pavilion, and open to the air on all sides, except where the bridges of communication connect the wards with the staircases. For administrative purposes the kitchen can be placed in the centre, and the whole establishment of the sick house arranged so as to minimise the time and strength of the staff, and to promote economy everywhere.

2. That the sick chambers proper are divorced from all the other buildings essential to the equipment of a clinical hospital, so that patients and everything that appertains to their treatment and welfare are placed under the most favourable conditions hygienically and scientifically, which it is possible to secure to them in the most perfect of modern hospitals.

The model hospital would contain 754 beds, and we have estimated the cost to be from £350,000 to £400,000 according to the requirements insisted upon by the acting Medical Staff. In any case a hospital can be erected on the pavilion plan at a less cost than that of any other plan yet prepared.

We think it due to Mr. I'Anson to express our appreciation of the great industry and devotion he has brought to bear upon the evolution of plans from the instructions of many councillors, all of whom were in search of a solution of difficulties of which most of them probably had but imperfect knowledge.

---

### DESCRIPTION OF MODEL PAVILION PLAN.

The new pavilion hospital would contain 750 beds, as follows:—(1) Medical beds in two pavilions of 5 stories each containing 10 wards of 24 beds, 240 beds. (2) Ten three-bedded wards in same pavilions unappropriated, 30 beds. (3) Surgical beds in two pavilions, each containing 5 stories, making 10 large wards of 24 beds, and 10 small wards of 3 beds, altogether 270 beds. (4) Separate pavilion, containing 140 beds in 10 wards of 14 beds each, assigned as follows:—Gynæcological: Three wards of 14 beds each, 42 beds; Ophthalmic: Two wards of 14 beds each, 28 beds; four surgical wards which could be used for special departments, 56 beds; one ward of 14 beds, or 8 beds in smaller wards if desired to be assigned, say 8 beds; 10 casualty beds; Isolation block, 70 beds; total, 754 beds.

This plan will provide for the gradual and ultimate rebuilding of the hospital so as to make it a perfect modern hospital. It retains the north wing and the great hall, with the present church and gateway. The wards in each pavilion consist of ground, first, second, third, and fourth floors.

The ward unit will contain every modern requisite, including:—Clinical laboratory, sisters' room, ward kitchen, accommodation for food, linen and patients' clothes, etc., nurses' W.C., patients' bath and lavatory, patients' W.C., sink room.

Each pavilion will have its own lift, and the whole of the pavilions will be in communication by a ground floor corridor running east and west, connecting every pavilion, so that the maximum of economy and convenience in administration and working is secured.

Throughout the pavilions, with the exception of the isolation block, the wall space per bed will be 10 feet; the superficial floor space per bed is 130 feet; the cubic space per bed is 1,625 cubic feet. The dimensions of the 24-bed wards are:—Length, 125 feet; breadth, 26 feet; height, 12 feet 6 inches.

The height of each pavilion will be, if raised on arches or piers, about 75 feet. Each pavilion will be self-contained and open on all sides to the free current of air, with the exception of the space occupied by the connecting bridges on each floor. The accommodation for the out-patients'

department and the pathological block will be in all respects equal to that provided for in Plan No. 9.

At the south end of each pavilion is situated a balcony on every floor, where the patients can be placed in fine weather.

The operation theatres (five in number) are situated at the northern end of each surgical pavilion, and are provided with a good north light, and with a skylight to such theatres as the surgeons may desire. There are also five clinical laboratories alternating with the theatres for the surgeons' use.

Each pavilion is provided with a lift and staircase, and in addition fire-escape staircases will be placed at the south end of every pavilion in connection with each ward.

The pavilions in which the medical wards are placed are planned so as to make them as perfect as possible for the requirements of the medical school.

The out-patients' department is placed at the corner of Little Britain and West Smithfield, being the north-east corner of the site, and will contain the dispensary and ten casualty beds, with ample accommodation for the resident medical officers.

The matron, vicar, steward, and the clerk of the works are provided with accommodation to the south of the out-patients' department.

The isolation block, containing 70 beds, has been placed to the south of the out-patients' department, between it and the north-east block of wards. Block "G" in the isolation pavilion in plan No. 9, has been adopted bodily for the purposes of this block plan.

ST. BARTHOLOMEW'S HOSPITAL.

Plan of Rebuilding on Pavilion principle to contain 754 Beds.

| | |
|---|---|
| That is Medical 10 Large Wards .. .. | 240 |
| Surgical 10 Large Wards and 10 3-Bed Wards .. | 70 |
| EXTRA BEDS. | |
| MEDICAL PAVILIONS. | |
| 10 3-Bed Wards .. .. .. | 30 |
| GYNÆCOLOGICAL, ETC, BLOCKS. | |
| Gynæco ogy 28—42 Beds .. | 42 |
| Ophthalmic .. .. .. .. .. | 28 |
| Surgical or Special Dep rt-ments .. .. .. .. .. .. | 53 |
| One B.d in 8 Small Wards Unassigned.. .. .. .. .. | 8 |
| Isolation Block .. .. .. | 70 |
| Casualty Beds in O.P. Department 6 or 10 .. .. | 10 |
| Total .. .. .. .. | 754 |

provided with 10 clinical laboratories, some of which, if desired, can be used as day rooms for the patients.

In the gynæcological, etc., block, special operating theatres are provided, so that each surgeon may have the fullest facilities for his work.

The kitchen has been placed on the north side of the connecting corridor, where provision has been made for the stores and steward's department, and a residence provided for the domestic servants. This arrangement will secure that the patients in every ward shall be supplied with hot meals certainly and promptly.

The pathological department, including inquest room and mortuary, post-mortem room and laboratories, is placed to the right of the gateway from West Smithfield.

The library, anatomical and medical theatres, and the dissection room, occupy positions to the south-west of the pathological blocks. They will be very spacious and specially planned so as to make them as perfect as possible for the requirements of the medical school.

It is believed that this plan makes provision for every requirement of the medical staff, and it has been so designed as to be capable of modification or adjustment so as to fulfil the exact conditions which the acting medical staff may consider necessary or desirable.

It will further be seen that this pavilion plan will provide St. Bartholomew's Hospital with entirely new buildings, with the exception of the ancient gateway, the Church, and the north block which are retained intact. The plan further has the advantage of securing the best aspect, that is, the only proper aspect which modern hospital wards and modern theatres ought to possess.

It is further economical, in the sense that it can be erected and completed at a smaller cost than any other perfected plan for a new hospital which has yet been devised. It also preserves the historical Church and north wing, the two features of St. Bartholomew's Hospital which are calculated to grow in interest as the years pass.

## Figures and Tables

**Figure f1:**
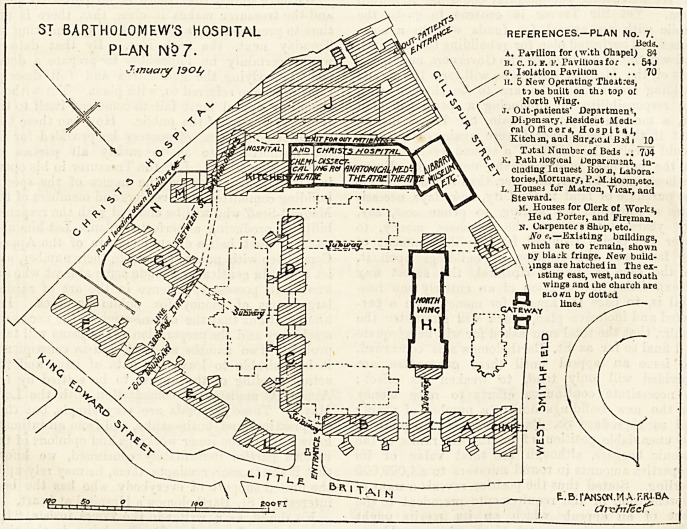


**Figure f2:**
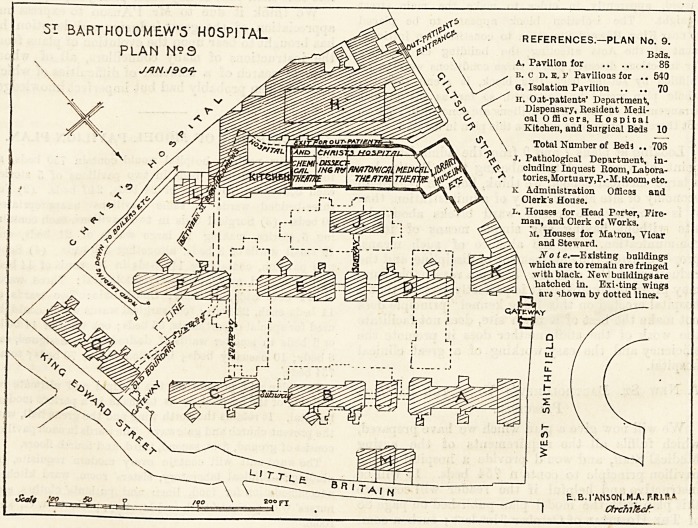


**Figure f3:**